# Opportunities for vein‐to‐vein datasets from a blood establishment perspective: Towards a ‘Pan‐European Transfusion Research InfrAstructure’

**DOI:** 10.1111/vox.70340

**Published:** 2026-08-18

**Authors:** Sophie M. T. Wehrens, Mikko Arvas, Suzanne F. Fustolo‐Gunnink, Matea Vinković Vlah, Allison Waters, Christian Erikstrup, Louise Ørnskov Drechsler, Simon J. Stanworth, Katja van den Hurk

**Affiliations:** ^1^ Department of Research Sanquin Blood Supply Foundation Amsterdam The Netherlands; ^2^ Research and Development, Finnish Red Cross Blood Service Helsinki Finland; ^3^ Paediatric Haematology, Amsterdam UMC Amsterdam The Netherlands; ^4^ Department of Research and Development Croatian Institute of Transfusion Medicine Zagreb Croatia; ^5^ Irish Blood Transfusion Service Dublin Ireland; ^6^ UCD School of Public Health, Physiotherapy and Sports Science University College Dublin Dublin Ireland; ^7^ Department of Clinical Immunology Aarhus University Hospital Aarhus Denmark; ^8^ Department of Clinical Medicine Aarhus University Aarhus Denmark; ^9^ NIHR Blood and Transplant Research Unit in Data‐Driven Transfusion Practice, Nuffield Division of Clinical Laboratory Sciences, Radcliffe Department of Medicine University of Oxford Oxford UK; ^10^ NHS Blood and Transplant Oxford University Hospitals NHS Foundation Trust and University of Oxford Oxford UK; ^11^ Public and Occupational Health, Amsterdam UMC Amsterdam The Netherlands; ^12^ Amsterdam Public Health Research Institute, Amsterdam UMC Amsterdam The Netherlands

**Keywords:** blood establishment, donor health, infrastructure, transfusion, vein‐to‐vein

## Abstract

**Background and Objectives:**

The ‘Pan‐European Transfusion Research InfrAstructure’ (PETRA) project was established to advance the use of donor, blood product and patient datasets in Europe, aiming to benefit both patient and donor health. Here, the initial PETRA objective was to describe the landscape of existing donor and blood establishment (BE) databases.

**Materials and Methods:**

An online survey was circulated to the European Blood Alliance's BE‐members. The survey collected information on the feasibility of accessing donor data, and challenges and possibilities for linking these datasets with information on the associated blood products and transfusion recipients, and donors' own health records.

**Results:**

Seventeen BEs across 16 countries completed the survey. The majority could, in principle, link their donor data to product data (13 BEs, 76%) and recipient data (10 BEs, 59%) for research purposes. However, capabilities were limited and in only 5 of 17 BEs was the donor to recipients' linkage an automated process. BEs reported significant challenges to achieve full vein‐to‐vein linkage, including legal constraints and lack of consent (11 BEs) and resources (10–14 BEs). Information technology and data issues as well as lack of knowledge and training were cited as obstacles by a minority of BEs.

**Conclusion:**

Whilst the survey results suggest considerable interest in developing linkages between blood donors, their products and recipients, many challenges remain due to a variety of obstacles. First steps in working towards a PETRA may be assistance to navigate legal frameworks as well as investing in resources and quality and harmonization of data collections.

**Highlights**

- Seventeen blood establishments (BEs) in 16 countries responded to a survey on obstacles and opportunities for achieving vein‐to‐vein datasets.
- In 10 (59%) of the BEs, donor‐to‐recipient links could be established for research improving transfusion outcomes, but only in 5 (29%) this was an automated process.
- In order to work towards a ‘Pan‐European Transfusion Research InfrAstructure’ (PETRA), legal frameworks, adequate donor consent and (financial and human) resources are the most common obstacles that require addressing.

## INTRODUCTION

Blood establishments (BEs) and hospitals across Europe hold significant amounts of data on donors, blood products and patients receiving these products. Better access to, and integrated analysis of, these data could lead to substantial advancements in transfusion medicine and donor health management. In some countries, these datasets are both well‐populated and well‐integrated into the healthcare infrastructure, such as in the Scandinavian Donation and Transfusion (SCANDAT) database [1]. However, in most countries and regions, donor and patient transfusion‐related datasets are scattered, and many do not link the donor to the product and the recipient for benchmarking or research purposes. Vein‐to‐vein datasets are areas of intense research interest internationally, as shown in the Recipient Epidemiology and Donor Evaluation Study (REDS) programme [2, 3] and roadmaps for donation and transfusion databases in Canada (CANDAT) [4] and Australia [5].

Therefore, we established a European collaborative project to work towards a ‘Pan‐European Transfusion Research InfrAstructure’ (PETRA), aiming to develop a strategic plan for further alignment of existing databases, methodologies and research efforts. The aim of this first step towards PETRA was to describe existing databases on donors across European countries, including possibilities and barriers for accessing these data and for establishing the vein‐to‐vein link between donor, product and the recipient.

## MATERIALS AND METHODS

To gain insight on current capabilities and limiting factors for the development of integrated donor, product and patient datasets among European BEs, an online survey was designed by an international interdisciplinary working group including specialists in donor and transfusion medicine research. The survey was created in Qualtrics XM, keeping in mind the heterogeneous settings of the BEs and possible responses they may (be able to) provide. Questions were made compulsory in order to achieve maximum sample size and representativeness for all questions.

In brief, the survey asked BEs about the feasibility of accessing donor data and the challenges and possibilities for linking these data to information on blood products and their recipients, as well as to the donors' own health records. A full copy of the survey is available in Data S2. Where BEs were asked about linking donor data to transfusion recipient data, we interpreted this as the vein‐to‐vein (v‐v) link and refer to these BEs, as v‐v BEs. Some questions (obstacles and plans to link to clinical databases) were asked separately in the setting of transfusion research on one hand and donor health research on the other hand as the databases, linkages and obstacles were expected to be different.

The survey was pre‐tested and piloted within the working group and by working group colleagues who provided feedback on question clarity, response options and survey flow. The survey was then circulated by the European Blood Alliance (EBA) in May 2024 to its 29 member countries via email (a list of countries is available in Methods S1). BEs received this email either directly from the EBA or through intermediate bodies or committees, especially for the countries where blood services operate regionally. BEs were reminded by the EBA and working group to complete the survey and the survey was closed from October 2024. Answers that were clearly incorrect were checked with the survey respondent and corrected. All respondents gave consent for their BE's data to be included in the inventory.

## RESULTS

The survey was completed by respondents from 17 BEs in 16 countries. They held data of, on average, 259,130 donors (total 4,405,209; median 62,000; range 12,188–1,409,411), making an average of 775,568 donations (total 13,184,648; median 98,901; range 15,829–7,455,040, a mix of donation types) in the year 2023. This number of donations comprised approximately 80% of the 16,725,892 successful donations (according to latest known data) made in the 29 EBA member countries. Eight BEs operate at a national level only and (apart from one) account for (nearly) all of the national donor information. Three organizations cover only a small percentage (1%–10%) of the national number of donations, reflected in the fact that two BEs are active at regional/local level and one at regional/local as well as national level.

Most organizations agreed that linking different steps in the donor‐product‐recipient chain is possible; that is, donor data can be linked for research purposes to product data by 13 BEs (76%) and to recipient data by 10 BEs (59%), investigating areas such as improving product quality and transfusion outcomes (Figures 1 and 2). However, the majority indicated that their organization does not have fully automated processes in place to achieve linkage, illustrated by the finding that automated linkage for haemovigilance and of donors to recipients was feasible in five BEs (29%) and of products to recipients in seven BEs (41%). Linking donors to products is automated in more, namely 10 (59%), of the BEs. Lack of automated processes was particularly the case for the steps that involve patients and linking their data to products and donors to improve research into transfusion outcomes.

**FIGURE 1:**
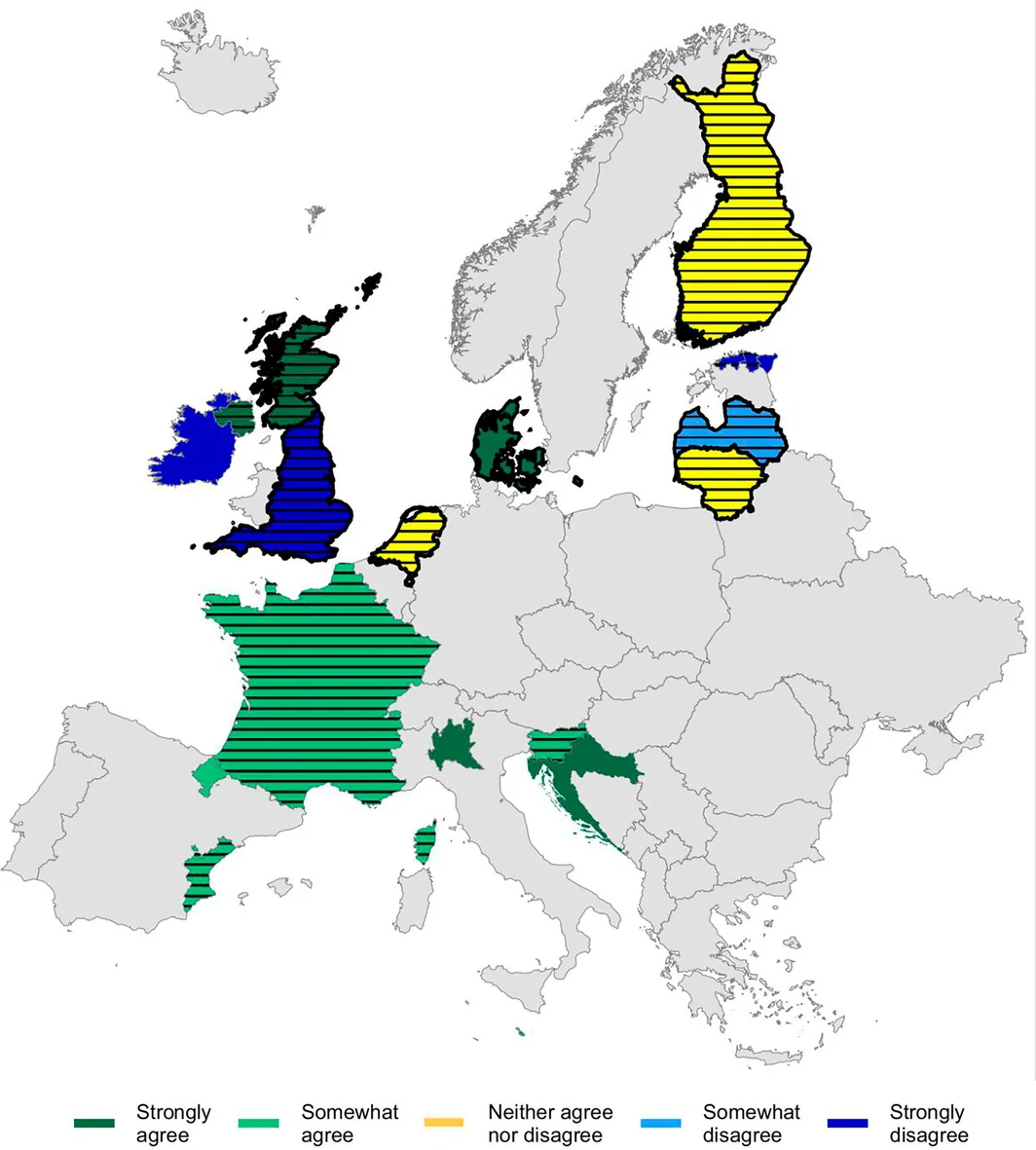
Map of Europe summarizing the linkage possibilities for the responding blood establishments (BEs). Countries with a black outline indicated that they represent their whole country and they have no (additional) regional/local cover. Colours indicate to what extent respondents agreed with the statement that linking donors to recipients for research purposes is possible: strongly agree (dark green), somewhat agree (light green), neither agree nor disagree (yellow), somewhat disagree (light blue) and strongly disagree (dark blue). Horizontal lines indicate countries/regions that had no fully automated process for linking donors and recipients (somewhat or strongly disagreed with the statement on fully automated linkage).

**FIGURE 2:**
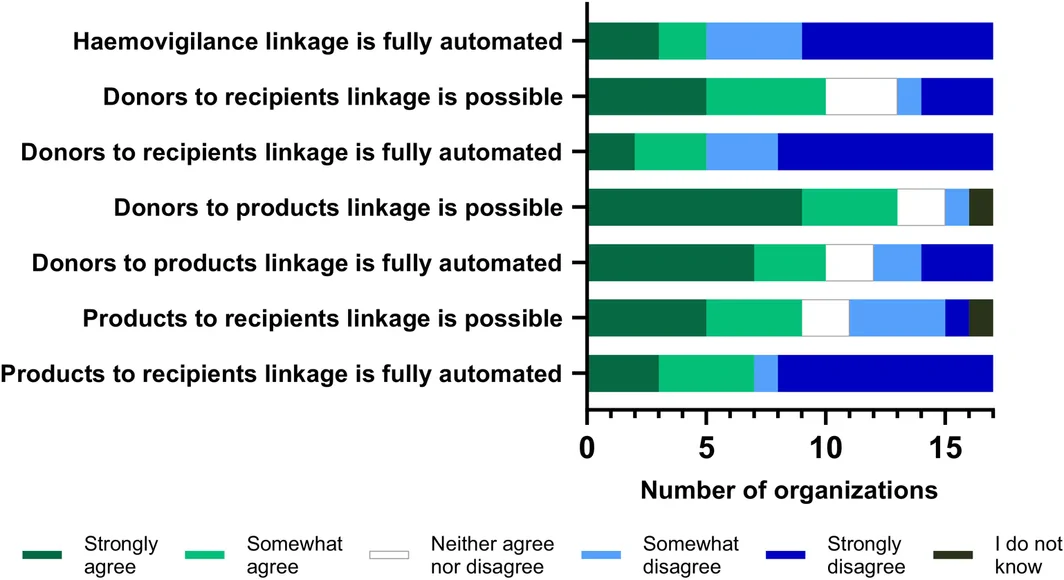
Extent to which blood establishments (BEs) agreed on seven statements asking whether different links in the vein‐to‐vein chain are currently possible with their database and whether this linkage is automated. The first statement refers to linking for legal haemovigilance purposes, the other six statements ask about enabling linkages for research purposes, for example into improving transfusion outcome and product quality.

Of the 17 BEs, 65% responded with ‘yes’ or ‘maybe’ when they were asked whether they currently have the ability or have any plans to link their donor/donation database to any existing or future clinical database (Figure 3). Six BEs answered this question on linkage with transfused patients with ‘yes’ and five with ‘maybe’ (Figure 3a). Similarly, four BEs (plan to) have and seven may have the ability to link their donor/donation database to the donors' own health records for research into donor health (Figure 3b).

**FIGURE 3:**
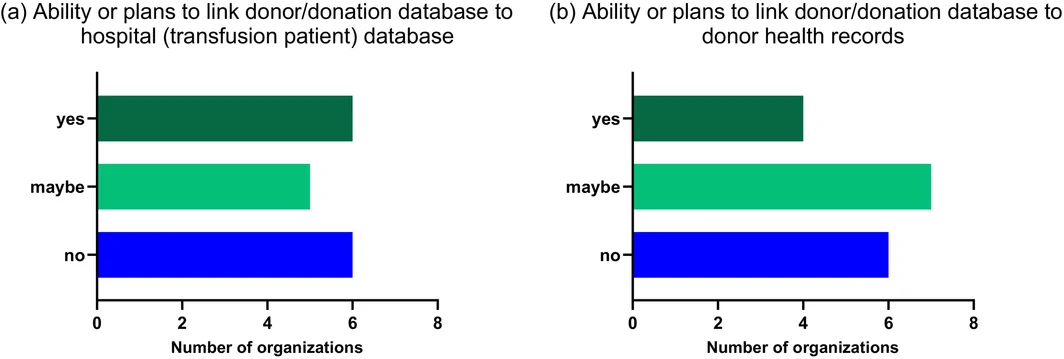
Number of blood establishments (BEs) that have the ability or have any plans to link their donor/donation database to any existing or future patient or hospital database in order to assess transfusion outcomes (a) or existing or future (hospital) database that holds the donor's own health records in order to follow up or research donor health (b).

Eleven (65%) of all BEs reported that legal constraints and lack of consent were extremely or somewhat likely obstacles to achieve donor‐to‐patient linkages to facilitate transfusion or donor health research (Figure 4a,b). Five (50%) of the 10 BEs that reported they could link donor to recipient data (v‐v BEs) stated legal constraints and lack of consent as likely obstacles (data not shown). Lack of staff or financial resources was cited as a likely obstacle for 14 (82%) of all BEs or seven of the v‐v BEs when using these databases to support transfusion research and by 10 (59%) of all BEs or four of the v‐v BEs to facilitate donor health research. Difficulty in finding variables in or matching between datasets was indicated by nine BEs (53%), four of the v‐v BEs, as a likely obstacle for donor health research and lack of compliance in completing records by nine organizations (53%), three of the v‐v BEs, for transfusion research. Information technology (IT) issues, lack of knowledge and training and the absence of a unique identifier were cited as specific research obstacles in a minority (less than seven) of BEs.

**FIGURE 4:**
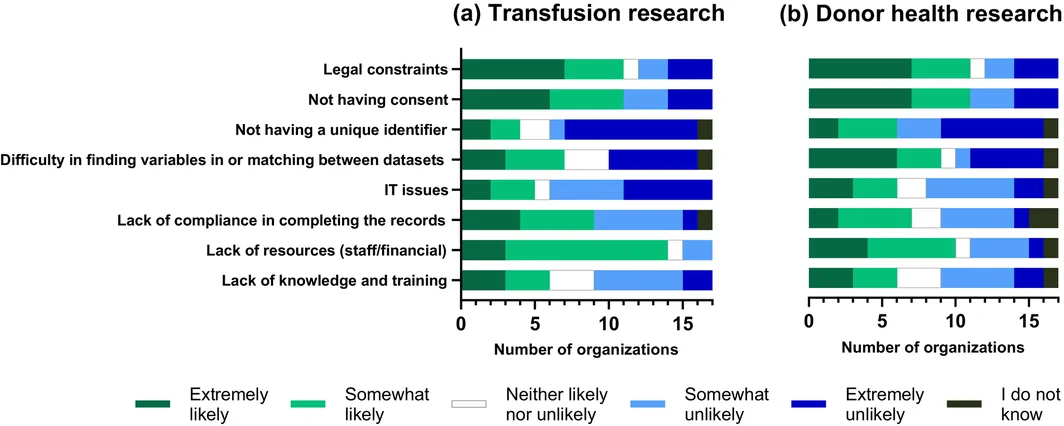
Likelihood that blood establishments (BEs) will encounter or observe eight different issues or obstacles when linking or trying to link donor, product and patient data for research into transfusion outcomes (a) or donor health (b). For some of the statements organizations were provided with examples (see Methods S2). IT, information technology.

## DISCUSSION

This study demonstrated substantial variability in the capabilities of 17 BEs in 16 European countries to access and connect donor, product and recipient data. Only 59% of BEs were (in principle) able to establish v‐v linkages, and just 29% could do this in a fully automated way. The results provide information on barriers for the creation of v‐v infrastructures that would allow for the conduct of high‐quality and efficient clinical transfusion and donor health research, as well as optimal quality control and stock management for BEs and hospitals.

Obstacles for further establishment of linkages were mainly legal constraints, not having donor consent and lack of financial and personnel resources. Interestingly, in those BEs that were able to establish the link between donors and recipients, obstacles were still observed and the trend was similar in terms of the percentage of BEs that reported the obstacles for the two different purposes (transfusion and donor health research). As BEs were asked if they would encounter *or* observe these issues/obstacles when linking *or* trying to link data, it may imply that both groups of BEs recognized these obstacles in a similar way but that those who had successfully established the link, had overcome these obstacles. In order to keep the survey short, no questions were added to assess the effort it took BEs to establish the linkages. However, ongoing conversations indicate that many organizations need to spend considerable time and resources on linking, even though they are (ultimately) able to establish one. In line with our findings, other roadmaps to clinical and v‐v datasets have also suggested that challenges in the ethical‐legal, funding and data quality areas as well as other barriers need to be overcome to reach successful implementation [4, 6, 7]. Of note for the interpretation of the current results as well as future steps, is the likely interdependency between obstacles; that is, investing in one (for example data harmonization) may resolve or improve another obstacle (such as lack of resources and knowledge). Once established, however, the potential of such datasets for advancing clinical and research priorities is evident [1, 2, 3, 7, 8]. Those integrated datasets enable and improve, for example, clinical practice such as clinical audit and feedback [6], surveillance for unknown transfusion‐transmitted pathogens [9] or studies into donor and component factors affecting recipient outcomes [10, 11, 12].

Contrary to the expectation that the different setting for transfusion compared to donor health research would result in different obstacles, observed barriers were similar in both settings, although lack of resources was more frequently reported to hinder linking for transfusion than for donor health research. On the other hand, plans to link donor/donation databases to clinical databases may be slightly more concrete in the field of transfusion than in donor health research.

There are strengths and limitations to our survey. A consideration in the interpretation of the results is the geographical spread and heterogeneity (e.g., in national vs. regional organization of BEs) in respondents. Although we did not systematically invite BEs to respond to the survey, this spread may have resulted in a good degree of representation. On the other hand, heterogeneity also made it difficult to design a survey that would fit all BEs. For example, a more precise estimate of the extent to which the BEs were able to establish linkages and which specific parts are automated (if not done fully) cannot be derived from the current questions and may have been interpreted differently by different BEs.

Our findings suggest that investment in resources as well as assistance to navigate legal frameworks including possibilities and exemptions under the General Data Protection Regulation (GDPR) and adequate (modular) informed consent may improve the potential to link these existing databases. Nearly all BEs were interested in further investigating possibilities to map and improve integration of donor, donation and patient data for transfusion and donor health research. Some BEs have already made changes following the completion of the survey.

The findings of this survey will enable collaborative development of a strategic plan for a Pan‐European transfusion infrastructure. Work to improve these areas will involve standards and recommendations in line with European regulations such as the GDPR, European Health Data Space (EHDS), Substances of Human origin (SoHo) and guidelines from the European Directorate for the Quality of Medicines & HealthCare (EDQM). Ultimately, these infrastructures will enable further research aimed at better donor health management, monitoring blood component utilization and improving patient outcomes.

## CONFLICT OF INTEREST STATEMENT

Christian Erikstrup has received unrestricted research grants from Novo Nordisk (administered by Aarhus University) and Abbott Diagnostics (administered by Aarhus University Hospital). He has received no personal fees.

## Supporting information


**Methods S1.** Member countries of the European Blood Alliance receiving the survey in May 2024, listed in alphabetical order.

**Methods S2.** PETRA—Donor databases.

## Data Availability

Data related to this publication are held at Sanquin Blood Supply Foundation. Requests to access these data in line with relevant regulations and agreements may be made to the corresponding authors.
