# Supplementary material for: Opportunities for vein‐to‐vein datasets from a blood establishment perspective: Towards a ‘Pan‐European Transfusion Research InfrAstructure’
Source: Vox Sang. 2026 Aug 18;121(11):1719–25. doi: 10.1111/vox.70340 (PMC13748350; doi:10.1111/vox.70340)
Supplement: Supplementary file 1 [file VOX-121-1719-s001.pdf]

PETRA - donor databases

Start of Block: Intro

Q1.1 Dear colleague,

Welcome to this survey towards a **Pan-European Transfusion Research infrAstructure (PETRA),** a project funded by the European Blood Alliance, led by Sanquin Blood Supply Foundation. We would be very grateful for your support and responses to this short survey aimed at understanding the capabilities of donor databases.

The application of data science in health research offers significant opportunities in transfusion medicine. There are also opportunities with better linkage of donor and patient data and alignment of infrastructure across Europe. The overarching aim of this PETRA project is to inform a strategic plan to direct further research at the European level, including aligned 'vein-to-vein' research infrastructures.

In this survey, we are also are seeking your interest in joining our project group to explore the feasibility of electronic data collection to efficiently understand ‘where blood goes’, and to investigate a clinical research question related to a select number of patient groups that are potentially over- or under-transfused.

The aim of this survey is to obtain an overview of the existing electronic platforms and datasets used in the blood supply chains across Europe. The survey will collect background and meta-data on existing donor- and product datasets. In a later survey we will approach organisations that hold clinical or patient datasets that might be relevant to blood transfusion. The results will be used to produce an inventory of European blood donor-, product- and transfusion databases as a basis for the strategic plan for a pan-European research infrastructure.

- Please note that given the nature of the survey, there may be a need to collect answers on certain parts of the survey from other individuals in your organisation or different colleagues. Rather than forwarding the survey for others to add to, we recommend that one person collects all answers and keeps track of survey completion. Even partially completed, your answers will be saved, so you won't have to take the survey from the beginning all over again if you need to pause. If you wish to do this, please make sure you use the same internet browser on the same device.
- If you wish to download a PDF-ready-to-print-version of this survey before completing it online, you can click on the link to the PETRA survey. You will also be able to view and print an overview of your responses before submitting them.
- We will process any personal data you provide in line with Sanquin's Privacy Statement. By completing this survey you consent for metadata about your dataset and organisation

to be mentioned in the inventory. We will only collect aggregated data about donors, products and patients and therefore donors and patients will never be identified in outcomes of the PETRA inventory, reports and publications.

- We estimate that filling out the survey takes around 30 minutes, depending on whether you have to collate the information, and you have up to 26th of May 2024 to complete this survey.

If you have any questions beforehand or while you are filling out the survey, please contact the PETRA team via e-mail at petraproject@sanquin.nl.

Thanks in advance for your time and effort!

On behalf of the PETRA team,

Dr. Sophie Wehrens

Dr Katja van den Hurk, Dr Suzanne Fustolo-Gunnink and Dr Sophie Wehrens (Sanquin Blood Supply Foundation, The Netherlands)
Prof Christian Erikstrup and Louise Ørnskov Drechsler (Aarhus University Hospital, Denmark)
Matea Vinković (Croatian Institute of Transfusion Medicine, Croatia)
Prof Simon Stanworth (European Haematology Association Specialised Working Group in Transfusion, the NIHR Blood and Transplant Unit in data, Oxford, UK)
Dr Mikko Arvas (Finnish Red Cross Blood Service, Finland)
Dr Allison Waters and Eileen Ryan (Irish Blood Transfusion Service, Children's Health Ireland, Crumlin Hospital, Ireland)
Prof Emanuele Di Angelantonio (NHSBT, University of Cambridge, UK)

End of Block: Intro

Start of Block: Consent organisation inventory

Q2.1 **Do you consent for your organisation's name and website to be listed in the PETRA inventory?**
Please note that if you select 'no', you can no longer complete the survey.

○ Yes

○ No

End of Block: Consent organisation inventory

Start of Block: Consent contact person inventory

Q3.1 **Do you consent for your name and contact details to be listed in the PETRA inventory?**
Your organisation will be mentioned in the inventory by default.

○ Yes

○ No

End of Block: Consent contact person inventory

Start of Block: Survey start

You can now start completion of the survey. For your information, you can still access the link to the survey pdf whilst completing it; it includes the instructions as well as all the questions. You will also be able to (re)view and print your responses before submitting them.

End of Block: Survey start

Start of Block: Contact person

Q4.1 **What is your name?**
i.e. the person providing the information for this survey and whom we may contact if we have any questions about your replies.

____

Q4.2 **What is your e-mail address?**
We will only use this e-mail address to ask for clarification on the answers provided (if necessary) and to share the results with you.

____

End of Block: Contact person

Start of Block: Organisation

Q5.1 **What is the name of the primary organisation that you represent?**

____

Q5.2 **Which of the descriptions below best describes your organisation?**
select all that apply

- ▢ Blood collection service
- ▢ Blood product processing and release
- ▢ University/Academic hospital
- ▢ General hospital
- ▢ University
- ▢ Research (Institute)
- ▢ Other, please specify ____

Q5.3 **What is your organisation's website?**

____

Page Break

Q5.4 **In what country is your organisation active?**
Please choose from the auto-complete list (country names are in English)

Q5.5 **Is your organisation active at national or regional level?**

- ○ National
- ○ Regional/local
- ○ Both national and regional/local

End of Block: Organisation

Start of Block: Population in your database

Q6.1 **The following questions are about the number of donors or donations your organisation holds information on. Please answer all questions; use whole numbers without decimal places only, enter '0' if your organisation does not hold this information or 'I do not know' if you do not know the percentage. Where we ask for the percentage we mean the number of donors or donations your organisation holds information on as a percentage of the national number of donors or donations; please indicate if you had to estimate this number. Please provide the numbers for the year 2023.**

Page Break

Q6.2 **How many donors does your organisation hold information on?**
Please include all donors that donate blood (products) for transfusion purposes. Please complete both fields below and provide the total number of individuals that made a donation in the year 2023.

☐ absolute number in the organisation (please use numbers only; enter 0 if your organisation does not hold donor information)

____

☐ percentage of total national number of donors who donated in 2023 (please use numbers only; enter 0 if your organisation does not hold donor information. Or 'I do not know' if you do not know this information)

____

☐ I/we estimated the percentage of the total national number above (please select if you *estimated* rather than *know* or *calculated* the exact percentage)

Page Break

Q6.3 **How many donations does your organisation hold information on?**
Please complete both fields below and provide the total number of donations in the year 2023.

☐ absolute number in the organisation (please use numbers only; enter 0 if your organisation does not hold product/component information)

____

☐ percentage of total national number of donations (please use numbers only; enter 0 if your organisation does not hold product/component information. Or 'I do not know' if you do not know this information)

____

☐ I/we estimated the percentage of the total national number above (please select if you *estimated* rather than *know* or *calculated* the exact percentage)

Q6.4 Please explain how you have defined a donation for this survey:

○ We have included all *succesful* donations

○ We have included all donation *attempts*

○ We have used another defenition / selection criteria, namely:

____

Q6.5 Are there any particulars that we should know about the selection or inclusion criteria that you used to provide the numbers of donors and/or donations above in order to interpret these numbers?

____

____

____

____

End of Block: Population in your database

Start of Block: Electronic implementation

Q7.1 **The following questions assess electronic systems and implementation levels at your organisation.**

Page Break

Q7.2 Who is your electronic donor / blood bank management provider for your donor / donation database (for example eProgesa)?

- [ ] Our provider is (please specify software and current version in place): ____

- [ ] ⨂Our organisation does not have an electronic donor / blood bank management provider

- [ ] ⨂I do not know

- [ ] ⨂Our organisation uses manual processes in addition to an electronic blood bank system

Q7.3 Who is your electronic Laboratory Information Management System (LIMS) provider?

- ☐ Our provider is (please specify software and current version in place): ____
- ☐ ⊗Our organisation does not have a LIMS
- ☐ ⊗Data are fed back entirely into our donor database
- ☐ Data are partly fed back into our database, please describe: ____
- ☐ Data are not fed back into our donor database
- ☐ ⊗I do not know
- ☐ Our organisation uses manual processes in addition to an electronic laboratory system

Page Break

End of Block: Electronic implementation

Start of Block: Linkage and access

Q8.1 **The following questions aim to get an overview of the linkage and access possibilities that currently exist for donor/donation data and samples held by your organisation and any possibilities or obstacles you experience.**

Page Break

Q8.2 Do you contribute to a **national** database that holds a dataset containing predefined data types (either by directly adding donor and/or product data or by letting the central database copy or view data from your organisation's database)?

- ◯ Yes, please: (1) provide the name of the database, (2) specify whether this is a donor / product or combined database (one that also holds patient data) and (3) how often and when data accessible from this database are updated: ____

- ◯ No

Page Break

Q8.3 Please indicate to what extent you agree with the statements on your database and whether it currently supports a 'vein-to-vein' link from donor to product to a recipient database (e.g. at hospital level).

	Strongly disagree	Somewhat disagree	Neither agree nor disagree	Somewhat agree	Strongly agree	I do not know
Linking donors to products and patients for **haemovigilance** purposes is a fully **automated** process.	○	○	○	○	○	○
It is possible to link specific **donors** to **recipients**, e.g. for research into improving transfusion outcomes.	○	○	○	○	○	○
Linking specific **donors** to **recipients**, e.g. for research into improving transfusion outcomes, is a fully **automated** process.	○	○	○	○	○	○
It is possible to link specific **donors** to **products**, e.g. for research into improving transfusion product quality.	○	○	○	○	○	○
Linking specific **donors** to **products**, e.g. for research into improving transfusion product quality, is a fully **automated** process.	○	○	○	○	○	○

It is possible to link specific **products** to **recipients**, e.g. for research into improving transfusion outcomes.	○	○	○	○	○	○
Linking specific **products** to **recipients**, e.g. for research into improving transfusion outcomes, is a fully **automated** process.	○	○	○	○	○	○
We should be able to link transfusion outcomes to donor characteristics as we use a **unique product/donation identifier**.	○	○	○	○	○	○

Page Break

Q8.4 Can you or do you have any plans to link your donor/donation database to any existing or future *patient or hospital database* in order to assess *transfusion outcomes*?

○ No

○ Maybe, please provide, if possible, the name of the database, website and details of a contact person whom we may contact for further information: *Please ensure you have permission from this contact person and the owner of the database to provide us with this information* ____

○ Yes, please provide, if possible, the name of the database, website and details of a contact person whom we may contact for further information: *Please ensure you have permission from this contact person and the owner of the database to provide us with this information.* ____

Q8.5 **How likely is it that you will encounter or observe the below issues/obstacles for your organisation when linking or trying to link donor, product and *patient data for research into transfusion outcomes*?**

	Extremely unlikely	Somewhat unlikely	Neither likely nor unlikely	Somewhat likely	Extremely likely	I do not know
Legal constraints (for example General Data Protection Regulation (GDPR))	○	○	○	○	○	○
Not having consent	○	○	○	○	○	○
Not having a unique identifier	○	○	○	○	○	○
Variables are not easy to find within a dataset and/or do not match between datasets (e.g. because of different nomenclature or definitions)	○	○	○	○	○	○
IT issues (for example lack of electronic system)	○	○	○	○	○	○
Lack of compliance in completing the records	○	○	○	○	○	○
Lack of resources (staff and / or financial)	○	○	○	○	○	○
Lack of knowledge and training	○	○	○	○	○	○

Other issue, please specify:	○	○	○	○	○	○

Q8.6 If you cannot currently make the link, between donor, product and patient (recipient), do you have an idea which kind of solutions could enable this linkage in theory?

○ No

○ Yes, please specify: ____

Page Break

Q8.7 Can you or do you have any plans to link your donor/donation database to any existing or future (hospital) database *that holds the donor's own health records* in order to follow up or research *donor health*?

- ◯ No
- ◯ Maybe, please provide, if possible, the name of the database, website and details of a contact person whom we may contact for further information: *Please ensure you have permission from this contact person and the owner of the database to provide us with this information* ____
- ◯ Yes, please provide, if possible, the name of the database, website and details of a contact person whom we may contact for further information: *Please ensure you have permission from this contact person and the owner of the database to provide us with this information.* ____

Q8.8 **How likely is it that you will encounter or observe the below issues/obstacles for your organisation when linking or trying to link donor data to *donor health records for research into donor health*?**

	Extremely unlikely	Somewhat unlikely	Neither likely nor unlikely	Somewhat likely	Extremely likely	I do not know
Legal constraints (for example General Data Protection Regulation (GDPR))						
Not having consent						
Not having a unique identifier						
Variables are not easy to find within a dataset and/or do not match between datasets (e.g. because of different nomenclature or definitions)						
IT issues (for example lack of electronic system)						
Lack of compliance in completing the records						
Lack of resources (staff and / or financial)						
Lack of knowledge and training						

Other issue, please specify:	○	○	○	○	○	○

Q8.9 If you cannot currently make the link, between your donor data and donor health records, do you have an idea which kind of solutions could enable this linkage in theory?

○ No

○ Yes, please specify: ____

Page Break

Q8.10 **How is access to data/samples held by your organisation controlled?**
Here we mean access that is required to carry out activities that are directly related to safeguarding the transfusion chain (donors, blood products and recipients)

○ Internal access for all employees

○ Internal access for certain authorised departments or employees

○ Internal access plus sharing with specific external partners for health care (e.g. General Practitioner, medical specialist)

Q8.11 **How is external access to data/samples held by your organisation governed arranged for (clinical) research?**
Assuming that access for external parties for (clinical) research needs to meet certain

conditions can you select all conditions that may need to be met or checked or bodies/individuals that need to be consulted in order to gain access?

▢ Specific clinical / research department that holds the data/tissue

▢ Data/tissue access committee

▢ Internal governance approval (e.g. to check compliance and feasibility)

▢ External governance approval (e.g. to check compliance and feasibility)

▢ Internal ethical approval (e.g. Institutional Review Board, Medical Ethics Committee)

▢ External ethical approval (e.g. Institutional Review Board, Medical Ethics Committee)

▢ Assessment by an internal Data Protection Officer or Legal Counsel

▢ Other, please specify:
____

▢ ⊗All data that are available to external parties are open access

▢ ⊗I do not know

End of Block: Linkage and access

Start of Block: Next steps

X→

Q9.1 **Would you be interested in being involved in our Proof of Concept study ("where does blood go?")?**
Here we will asses feasibility of electronic data collection to investigate a clinical research question related to a select number of patient groups that are potentially over- or under-transfused. We would work together to create a small vein-to-vein database with actual patient/donor data, to see how that could be organised and scaled up in the future. If you select 'yes' or 'maybe' we may contact you via the email address provided earlier in this survey.

Whether we contact you or not may, for example, depend on capacity and requirements for the clinical question to be investigated.

○ Yes

○ Maybe

○ No

End of Block: Next steps

Start of Block: Comments

Q10.1 **Is there any information on: existing electronic platforms and datasets used in the blood donation and transfusion chains possibilities or challenges for linking these datasets that you would like to provide, which did not fit in the questions above?** *If so, please explain below:*

____

____

____

____

____

Q10.2 **Are there any comments you would like us to add to the inventory for your organisation?**

____

____

____

____

____

End of Block: Comments

Start of Block: Sample / data types

Q11.1 In the following questions we will ask about the data/samples that your organisation collects or holds in order to get an idea of the data types that can be linked. Please include data/samples that you include as part of the ***routine process*** during donor checks, blood collection or blood product processing/release in the majority of that group. We mean all measurements that are obtained under your current policy even if not applicable to every donor, donation, product or patient (e.g. ferritin measurement may be part of the current donor check but not be measured every donation; if this is the case, please still select this parameter).

These questions on data / samples are optional in order to save you time. If you do not wish to answer these questions, simply skip the questions by clicking the 'next' button.

Page Break

Q11.2 Which ***donor*** data do you collect / hold as part of the ***routine process****?*
Please select all options that apply to your organisation.

- ☐ my organisation does not collect / hold donor data or samples
- ☐ Age
- ☐ Weight
- ☐ Sex
- ☐ Other demographics
- ☐ Blood pressure
- ☐ Capillary hemoglobin - primary method for donor screening
- ☐ Capillary hemoglobin - secondary method for donor screening in specific cases
- ☐ Infectious disease screening
- ☐ Ferritin measurements
- ☐ Donation and deferral history
- ☐ Adverse reactions to blood donation
- ☐ Socioeconomic data (e.g. education, job type, income)
- ☐ Lifestyle data (e.g. physical activity, diet, smoking)
- ☐ Complete full blood count data
- ☐ Hemoglobin only - primary method for donor screening

- [ ] Hemoglobin only - secondary method for donor screening in specific cases
- [ ] Other or specific set of full blood count paramters, please specify:

- [ ] “Omics”, such as genomics data
- [ ] genetics - smaller datasets, such as genotypes or SNPs
- [ ] Clinical biochemistry
- [ ] Other laboratory measurements, please specify:

- [ ] Diagnosis codes
- [ ] Prescriptions
- [ ] plasma
- [ ] serum
- [ ] buffy coats
- [ ] peripheral blood mononuclear cells (PBMCs)
- [ ] DNA
- [ ] fresh samples
- [ ] involvement in research
- [ ] re-contact for further research
- [ ] linkage with health records

☐ other data or samples, please specify: ________________________________

Page Break

Q11.3 Which ***blood product and component data*** do you collect / hold as part of the ***routine process****?*

Please select all options that apply to your organisation.

- ☐ ⊗ my organisation does not collect / blood product or component data
- ☐ type of component
- ☐ the DATE at which different processing steps were carried out
- ☐ the TIME at which different processing steps were carried out
- ☐ quality control data
- ☐ other data, please specify: ____

Page Break

End of Block: Sample / data types

Start of Block: End of survey

Q12.1 **You have now come to the end of the survey. On the next page you will find a summary of your responses. You can still edit these by using the left arrows to go back.**

Once you click the right arrow ('next' button) below the summary, your responses will be recorded and you are no longer able to go back and edit them.

End of Block: End of survey
