# Supplementary material for: Opportunities for vein‐to‐vein datasets from a blood establishment perspective: Towards a ‘Pan‐European Transfusion Research InfrAstructure’
Source: Vox Sang. 2026 Aug 18;121(11):1719–25. doi: 10.1111/vox.70340 (PMC13748350; doi:10.1111/vox.70340)
Supplement: Supplementary file 2 [file VOX-121-1719-s002.pdf]

Member countries of the European Blood Alliance receiving the survey in May 2024, listed in alphabetical order

1. Austria
2. Belgium
3. Croatia
4. Denmark
5. Estonia
6. Finland
7. France
8. Germany
9. Greece
10. Iceland
11. Ireland
12. Italy
13. Latvia
14. Lithuania
15. Luxembourg
16. Malta
17. Netherlands
18. North Macedonia
19. Norway
20. Portugal
21. Serbia
22. Slovenia
23. Spain
24. Sweden
25. Switzerland
26. UK - England
27. UK - Northern Ireland
28. UK - Scotland
29. UK - Wales
